# Tislelizumab-induced hemophagocytic lymphohistiocytosis in a patient with microsatellite instability-high colon cancer and coexisting systemic lupus erythematosus: a case report and literature review

**DOI:** 10.3389/fonc.2025.1585133

**Published:** 2025-08-06

**Authors:** Pengqing Jiao, Song Wang, Jianhua Wu, Yufei Zhao, Jiaxu Han, Ziyue Sha

**Affiliations:** ^1^ Department of Immunology and Rheumatology, The Fourth Hospital of Hebei Medical University, Shijiazhuang, China; ^2^ Animal Center, The Fourth Hospital of Hebei Medical University, Shijiazhuang, China; ^3^ Department of General Surgery, The Fourth Hospital of Hebei Medical University, Shijiazhuang, China

**Keywords:** MSI-H, PD-1, colon cancer, SLE, HLH

## Abstract

Immune checkpoint inhibitors (ICIs) have profoundly transformed the treatment landscape for microsatellite instability-high (MSI-H) colorectal cancer (CRC). However, immune-related adverse events (irAEs) remain a common and unpredictable complication among patients undergoing ICI therapy. Hemophagocytic lymphohistiocytosis (HLH), a rare and life-threatening irAE, is triggered by hyperactivated immune cells and excessive secretion of proinflammatory cytokines. We report a case of an MSI-H female patient with a history of systemic lupus erythematosus (SLE) who developed HLH after treatment with the anti-programmed cell death protein 1 (PD-1) antibody tislelizumab. After treated with tislelizumab 31 days, the patient presented with fever, pancytopenia, hemophagocytosis in bone marrow, elevated ferritin and triglyceride levels, and decreased fibrinogen. A diagnosis of HLH was confirmed with an H-score of 264. Despite steroid therapy, the patient’s HLH progressed rapidly. Etoposide was deemed intolerable, and tocilizumab and immunoglobulin were declined due to financial constraints. Regrettably, the patient succumbed to HLH within 16 days of diagnosis. This is the first reported case of ICI-induced HLH in an MSI-H colon cancer patient with a history of SLE, prompting an analysis of the potential mechanisms underlying the induction of HLH in this case. Clinicians should be vigilant for the development of HLH during ICI treatment and initiate combination therapy as early as possible upon onset of HLH.

## Introduction

Colorectal cancer (CRC) is the third most common cancer worldwide with an estimated incidence of over 1.9 million new cases annually, and it is the second leading cause of cancer-related mortality with 904,000 deaths (1). Approximately 20% of patients present at an advanced stage without the opportunity for surgery (2). Fluorouracil-based and platinum-based combinations are frequently utilized as chemotherapeutic agents for these advanced patients (3). Targeted agents such as bevacizumab or cetuximab, depending on the RAS gene mutational status and tumor location, are combined to augment the anti-tumor effect of chemotherapy agents (4). Nevertheless, the prognosis for these patients remains bleak (5). Immune checkpoint inhibitors (ICIs) targeting the programmed cell death protein 1 (PD-1) and cytotoxic T-lymphocyte–associated protein 4 (CTLA-4) have made significant breakthroughs in cancer treatment. Following their initial approval for melanoma treatment, ICIs have been widely applied in adjuvant treatment for various types of cancer (6). The KEYNOTE-177 trial showed that an anti-PD-1 antibody of pembrolizumab significantly improved the progression-free survival (PFS) compared to chemotherapy (16.5 months versus 8.2 months) as a first-line treatment in advanced CRC patients with microsatellite instability-high (MSI-H) or mismatch repair-deficient (dMMR) (7). Based on the result of KEYNOTE-177, pembrolizumab was approved by Food and Drug Administration (FDA) as the first-line treatment for MSI-H advanced CRC patients.

ICIs are believed to primarily exert anti-tumor effects by activating immune responses, particularly T lymphocytes (8). However, as a result of antigens shared between the tumor tissue and the target organ, the activated immune response may lead to T cell cross-reactivity, thereby resulting in the development of immune-related adverse events (irAEs) (9). The incidence of irAEs for ICI treatment of MSI-H colorectal cancer is approximately 31%, with 9% classified as grade 3–4 irAEs. These frequently include hypothyroidism, colitis, hyperthyroidism, pneumonitis and adrenal insufficiency (7). Nevertheless, hemophagocytic lymphohistiocytosis (HLH) caused by ICIs in MSI-H colorectal cancer patients has not been reported yet.

HLH is a rare syndrome charactered by high fever, cytopenia, hepatosplenomegaly, elevated levels of liver enzymes, and high serum levels of ferritin and triglyceride (10). The disease commonly results from the hyper-activation of immune cells, leading to excessive pro-inflammatory cytokine secretion, progressive tissue injury, multi-organ failure, and ultimately mortality (11). HLH is classified as primary or acquired based on its etiology, with the latter typically triggered by malignancies, severe acute infections, autoimmune diseases and exposure to drugs including ICIs (12). A study indicates that 5.7% of all HLH cases are associated with the application of ICIs (13). However, cancer patients with ICIs-related HLH had a history of autoimmune disease were rarely reported. Here, we presented a case of HLH following treatment with an anti-PD-1 antibody of tislelizumab in a patient with MSI-H colon cancer and co-existing systemic lupus erythematosus (SLE).

## Case report

**Figure 1:**
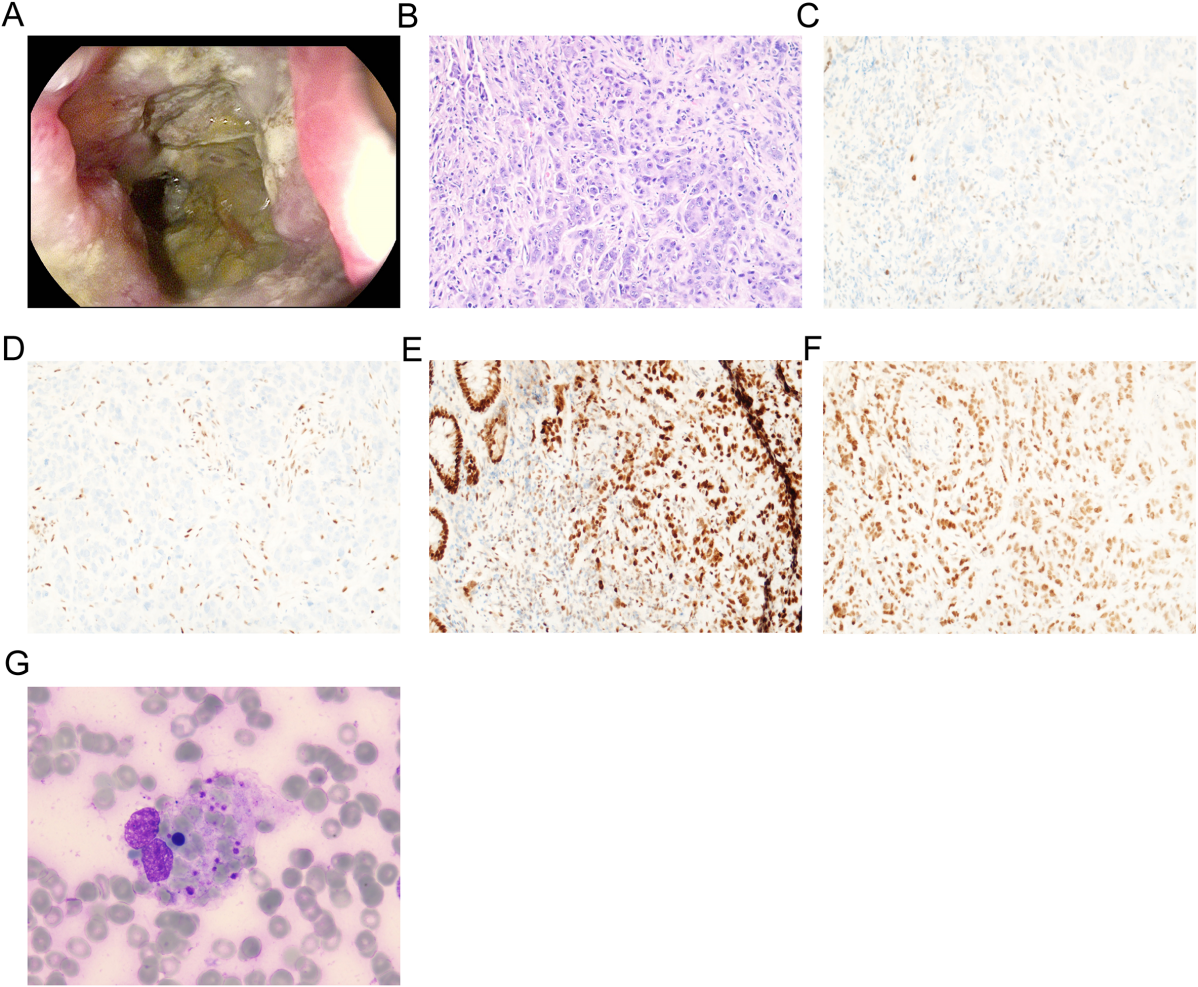
Gastroscopy and biopsy of the patient. **(A)** The colonoscopy examination revealed an ulcerative mass in the ascending colon; **(B)** The histopathological examination of biopsies sampled revealed poorly differentiated adenocarcinoma (stained using the H&E method, magnification, x200); **(C)** PMS2(-) by immunohistochemistry (magnification, x200); **(D)** MLH1(-) by immunohistochemistry (magnification, x200); **(E)** MSH2(+) by immunohistochemistry (magnification, x200); **(F)** MSH6(+) by immunohistochemistry (magnification, x200); **(G)** Bone marrow biopsy of the patient (magnification, x400).

**Figure 2:**
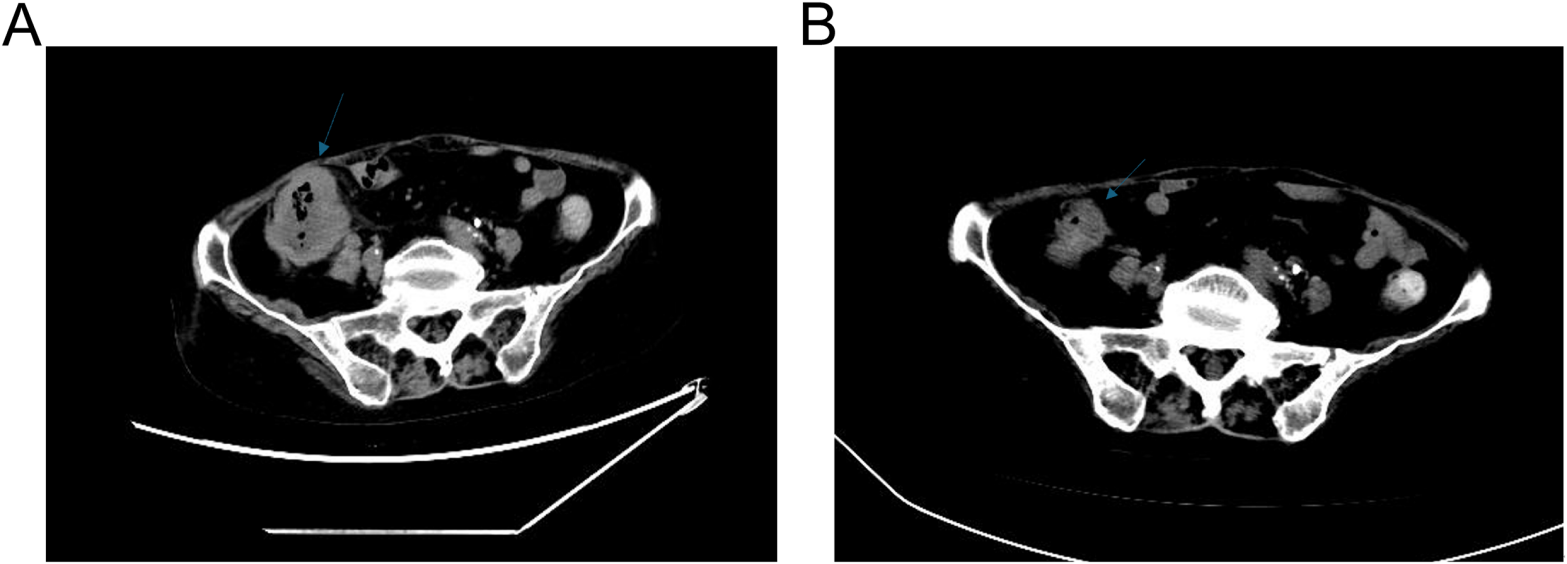
CT of the patient. **(A)** Abdominal CT scan prior to the treatment of tislelizumab. **(B)** Abdominal CT scan after the treatment of tislelizumab.

**Figure 3:**
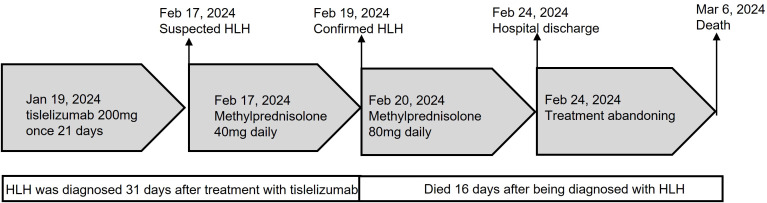
Treatment summary of the patient.

A 68-year-old Chinese female was admitted to the Fourth Hospital of Hebei Medical University on January 15, 2024, presenting with a complaint of appetite loss persisting for over a month. Notably, her medical history included a three-year diagnosis of SLE. Upon admission, the patient was administered a daily dosage of 3 mg of methylprednisolone and 0.1 g of hydroxychloroquine, maintaining her SLE in a stable state. Twenty days prior to admission, a colonoscopy revealed an ulcerative mass in the ascending colon, causing lumen narrowing (**Figure 1A**). Pathological and immunohistochemical analyses confirmed the tumor as a poorly differentiated adenocarcinoma (**Figure 1B**) with MSI-H, characterized by PMS2 (-), MLH1 (-), MSH2 (+), and MSH6 (+) (**Figures 1C–F**). An enhanced computed tomography (CT) scan demonstrated ascending colon wall thickening accompanied by enlarged peripheral lymph nodes (**Figure 2A**), alongside suspected malignant lesions and multiple metastatic nodules in the pelvic and abdominal cavities, leading to a diagnosis of Stage IV colon carcinoma. Laboratory tests indicated a negative anti-dsDNA antibody, while the patient tested positive for ANA (1:320) with TOPO I and anti-Sm antibody. Additional laboratory findings revealed complement C3 was 0.69g/L (normal range: 0.7-1.4g/L), complement C4 0.12g/L (normal range: 0.1-0.4g/L), white blood cells (WBC) 9.01×10^12^/l (normal range: 3.8-5.1×10^12^/l), hemoglobin 81.7g/L (normal range: 115-150g/L), platelets 238×10^9^/l (normal range: 125-350×10^9^/l), D-dimer 0.193 mg/l (normal range: <0.243mg/L), triglyceride 1.98 mmol/L (normal range: 0.3-1.7mmol/L), and C-reactive protein (CRP) 82.3mg/L (normal range: 0-6.0mg/L). Routine urine analysis was within normal limits. Based on the patient’s clinical manifestations and laboratory results, the SLEDAI-2000 score was calculated as 0, confirming her SLE was in a stable phase. Given the patient’s stable SLE, she received tislelizumab - the only PD-1 monoclonal antibody with government-sponsored medical insurance coverage for MSI-H solid tumor indications in China, (200 mg intravenously once every 3 weeks) on January 19, 2024, and was successfully discharged post-treatment. The patient returned to the Fourth Hospital of Hebei Medical University for the next treatment cycle on February 17, 2024. Since her last discharge, she experienced intermittent appetite loss and fatigue. Physical examination found no throat congestion or swelling, and cardiopulmonary and abdominal assessments revealed no significant abnormalities. The blood counts showed WBC was 3.23×10^12^/l, hemoglobin of 75 g/l and platelets 12×10^9^/l. Her D-dimer was 4.782 mg/l, triglyceride 2.78mmol/L, fibrinogen 0.81 g/L (normal range: 2.38-4.98g/L), ferritin ≥2000ng/L (normal range: 13.0-150), C3 0.46g/L, and CRP 71mg/L. A follow-up CT scan (**Figure 2B**) showed persistent thickening of the ascending colon wall, but with a reduction in the number of abdominal and pelvic nodules, suggesting a partial response to colon cancer treatment. However, due to the patient’s predisposition to HLH induced by ICI, administration of tislelizumab was discontinued. Although a bone marrow aspiration was proposed, the patient declined. Consequently, the patient was treated with daily 40 mg of methylprednisolone, along with platelet and red blood cell transfusions, and symptomatic and supportive care. Three days later, the patient developed a non-infectious fever, peaking at 38.5°C. Following repeated and emphatic recommendations, the patient underwent a bone marrow aspiration, revealing mixed anemia and an irritable bone marrow image with prominent hemophagocytosis (**Figure 1G**). Based on these findings, the patient met the diagnostic criteria for HLH-2004, with an H-score of 264. Given her poor physical condition, chemotherapy with etoposide and cyclophosphamide was deemed unsuitable, and she refused cytokine antagonists and immunoglobulin due to financial constraints. The patient was then treated with 80 mg of methylprednisolone daily for an additional four days, but her condition failed to improve. Ultimately, the patient opted to discontinue treatment and passed away ten days after discharge. The treatment process of the patient is depicted in **Figure 3**.

## Discussion

Patients with cancer and concurrent autoimmune diseases (ADs) including SLE, have historically been excluded from ICIs clinical trials. This exclusion primarily stems from concerns that ADs may be exacerbated, leading to an increased risk of severe irAEs. Nevertheless, in clinical practice, an increasing number of such patients are being encountered, and evidence suggests that ICIs treatment can be both safe and effective for them. A retrospective study evaluated the therapeutic outcomes of 85 individuals diagnosed with both cancer and ADs who received anti-PD-1/PD-L1 antibodies therapy. Their outcomes were compared with those of patients without ADs. Although a higher proportion of AD patients experienced irAEs of any grade (65.9% compared to 39.9%), no significant difference was observed in the incidence of grade 3–4 events between the two groups. Furthermore, no notable differences were identified in prognosis (14). Three additional studies have similarly demonstrated the safety of ICIs in patients with cancer and coexisting ADs. In these studies, only 20% - 40% of patients experienced worsening of their autoimmune conditions, and all adverse effects were manageable. Discontinuation of ICI therapy was rare, and tumor responses were reported (15–19). A systematic review evaluated the use of ICIs in 123 cancer patients with a history of ADs (20). Overall, 92 patients (75%) experienced irAEs. Among them, 50 patients (41%) had exacerbations of pre-existing ADs, 31 patients (25%) developed new irAEs, and 11 patients (9%) experienced both types of events.

Based on these findings, the following conclusions can be drawn: 1) Patients with ADs including SLE, experience a higher overall incidence of irAEs than those without ADs; however, there is no significant difference in the occurrence of high-grade (grade 3–4) events. 2) The most common types of irAEs involve exacerbations of underlying ADs. This may result from PD-1/PD-L1 inhibition-induced activation of T cells and other immune cells, which leads to the release of pro-inflammatory cytokines (21, 22), potentially triggering off-target inflammation and irAEs (23). 3) Flare-ups of ADs generally do not necessitate discontinuation of ICIs therapy and can be effectively managed. 4) The effectiveness of ICIs appears similar in patients with and without ADs (24). Despite this evidence, patients with cancer and ADs currently lack standardized treatment guidelines and reliable methods for assessing individual benefit-risk ratios. This gap represents a significant unmet need in oncology. Therefore, prospective studies are urgently needed to establish optimal strategies for administering ICIs in this patient population.

The H-score, which integrates various clinical indicators such as recognized immunosuppression, elevated temperature, and organomegaly; laboratory findings encompassing triglycerides, ferritin, serum glutamic oxaloacetic transaminase levels, fibrinogen levels, and cytopenia; as well as a specific cytological criterion (the presence of hemophagocytosis in bone marrow aspirate), is extensively employed for diagnosing HLH (25). A score exceeding 250, accompanied by a clinical presentation devoid of detectable infection, strongly indicates HLH. The patient exhibited a clinical profile characterized by fever, pancytopenia, hemophagocytosis in the bone marrow, elevated ferritin and triglyceride levels, and decreased fibrinogen, collectively pointing towards a diagnosis of HLH. The onset of HLH can be precipitated by multiple external factors, including malignancies, severe acute infections, autoimmune diseases, and exposure to ICIs (26). Hematological malignancies are the most common malignancies associated with HLH, whereas solid tumors are infrequently reported (27). Notably, no cases have established a direct link between colon cancer and the initiation of HLH.

During ICIs treatment, the inhibition of the PD-1/PD-L1 signaling pathway disrupts the normal inhibitory control that negatively regulates T-cell function. This leads to hyperactivation of cytotoxic T lymphocytes (CTLs) and excessive secretion of pro-inflammatory cytokines in healthy tissues (9, 28). HLH, a rare and severe irAE, can be triggered by hyperactivated immune cells and oversecretion of pro-inflammatory cytokines (12). Pathological features of HLH reveal abundant infiltration of immune cells, including CTLs and macrophages, along with oversecretion of pro-inflammatory cytokines such as interferon (IFN)-γ, tumor necrosis factor (TNF)-α, interleukin (IL)-1β, IL-6, IL-10, and IL-18 (29, 30). The molecular mechanisms underlying HLH are not fully understood. However, some studies suggest that excessive CTLs secrete significant amounts of IFN-γ, which potently stimulates macrophages. These macrophages then release large quantities of IL-1β, IL-6, IL-18, and TNF-α, potentially leading to tissue injury (31). Subsequent tissue damage produces high levels of IL-1β and IL-33, which continue to activate macrophages (30, 32). Additionally, IL-18, in combination with IL-12, is believed to facilitate further activation of CTLs and their production of IFN-γ (33, 34). On the other hand, IFN-γ enhances the expression of major histocompatibility complex (MHC) I on immune cells, thereby amplifying the feed-forward loops (12). This cytokine storm may exacerbate hematopoietic failure, contribute to various clinical manifestations of HLH, and worsen the disease (31). An HLH animal model suggests that IFN-γ contributes to anemia via inhibiting erythropoiesis and promoting hemophagocytosis (35). Another animal model indicates that release of IFN-γ leads to splenomegaly in HLH (12, 36). Furthermore, ferritin secreted by macrophages elevates plasminogen activator levels and triggers hyperfibrinolysis (37). Other pro-inflammatory cytokines, such as TNF-α, IL-1β and IL-6, are thought to be associated with fever and multi-organ dysfunction in HLH (38). Considering the critical role of cytokines in the pathogenesis of HLH, we strongly recommended that the patient undergo cytokine testing at the time of HLA diagnosis. However, the patient’s family declined the examination for economic reasons, as it was not covered by government-sponsored medical insurance.

To evaluate the impact of ADs on the onset time of ICIs-related HLH, we identified 28 cases of ICIs- related HLH from 25 articles with sufficient clinical data. The cohort characteristics are summarized in **Table 1**. Our analysis reveals that 8 patients had concomitant ADs. The median time from ICI infusion to HLH onset was 59.5 days in patients without ADs, compared to 32 days in those with ADs, suggesting ADs may accelerate the occurrence of ICI-related HLH. This temporal disparity could be attributed to pre-existing cytokine storm and excessive immune cell infiltration in ADs (21, 22). Specifically, in SLE, impaired antigen clearance triggers aberrant activation of lymphocytes and macrophages, along with excessive pro-inflammatory cytokine production. Such a hyperinflammatory milieu primes the immune system, increasing susceptibility to rapid HLH progression upon ICI exposure (39, 40). Additionally, MSI-H status, a feature of this case, confers high tumor mutational burden due to defective mismatch repair. This generates abundant neoantigens that persistently activate CD8^+^ T cells and natural killer cells, sustaining immune hyperactivation and providing a pathological basis for HLH (41). The synergy between SLE-driven autoimmunity and MSI-H immunogenicity establishes a “dual-engine” immune stimulation mechanism. This likely ignited a cascading inflammatory response, culminating in fulminant HLH within 31 days in our patient. Despite these insights, our conclusions require cautious interpretation due to clinical heterogeneity: variations in ADs subtypes, malignancy types, and ICI regimens across the cohort may confound onset-time comparisons. Moreover, our patient’s non-adherence to regular hematological monitoring outside the hospital, along with initial refusal of bone marrow aspiration may obscure the true HLH onset timeline.

**Table 1:** ICIs-related HLH reported in the literature in patients with cancer.

Study References	Malignancy type and stage	Age and sex	ICI	ADs	Time To HLH (days)
(48)	RCC, IV	54, M	Nivolumab + ipilimumab	None	6
(49)	LUAD, IV	78, M	Pembrolizumab	None	7
(50)	LUSC, IIIB	78, M	Pembrolizumab	None	10
(51)	Melanoma, IV	35, F	Ipilimumab + nivolumab	None	21
(52)	NSCLC, IV	63, F	Nivolumab	None	22
(53)	Choroidal melanoma, IV	65, F	Ipilimumab	None	42
(54)	Glioblastoma	74, M	Nivolumab	None	47
(55)	Melanoma, IV	42, M	Ipilimumab + nivolumab	None	51
(56)	Melanoma, IV	52, F	Ipilimumab	None	56
(57)	LUAD, IIIB	52, F	Nivolumab	None	56
(58)	Melanoma, IV	57, F	Ipilimumab + nivolumab	None	63
(55)	Melanoma, IV	36, M	Nivolumab	None	78
(55)	Melanoma, IV	32, M	Ipilimumab + nivolumab	None	91
(59)	Breast cancer, IV	58, F	Pembrolizumab	None	93
(60)	Melanoma, IV	58, M	Pembrolizumab	None	136
(61)	NSCLC, IV	60, M	Pembrolizumab	None	186
(61)	NSCLC, IV	60, M	Pembrolizumab	None	210
(62)	Kaposi sarcoma	85, M	Nivolumab	None	240
(63)	Bladder, IV	76, M	Pembrolizumab	None	270
(64)	OPSCC, IV	61, M	Pembrolizumab	None	277
(47)	Thymic carcinoma, IV	50, F	Pembrolizumab	Sjögren’s syndrome	7
(44)	CESC, IVB	73, F	Pembrolizumab	Psoriasis	12
(65)	Melanoma, IV	69, F	Ipilimumab + nivolumab	Sarcoidosis	22
(66)	LUSC, IIIB	74, M	Pembrolizumab	Rheumatoid arthritis	27
(67)	LUAD, IV	65, F	Atezolizumab	Antinuclear antibody positive	37
(68)	Melanoma, IV	26, F	Ipilimumab + nivolumab	Immune thyroiditis	70
(45)	Gastric cancer, IV	79, M	Nivolumab	Ankylosing spondylitis	143
(46)	Thymic carcinoma, IV	49, M	Pembrolizumab	Psoriasis	365

ICIs, Immune checkpoint inhibitors; HLH, Haemophagocytic lymphohistiocytosis; M, Male; F, Female; RCC, Renal cell carcinoma; LUAD, Lung adenocarcinoma; LUSC, Lung squamous cell carcinoma; NSCLC, Non-small cell lung cancer; OPSCC, Oropharyngeal squamous cell carcinoma; ITP, Immune thrombocytopenic purpura; CESC, Cervical squamous carcinoma.

Although the Society for Immunotherapy of Cancer (SITC) classifies HLH as a fatal irAE, it fails to provide specific treatment recommendations (42). Typical management guidelines for HLH propose potential benefits from therapeutic interventions such as steroids, immunosuppressive agents like cyclophosphamide and etoposide, intravenous immunoglobulin, and the anti-IL-6 receptor antibody tocilizumab (12, 43). In this particular case, despite attempting steroid therapy, the patient’s condition rapidly deteriorated. Given the patient’s frail physical state, chemotherapy was deemed unfeasible, and etoposide was consequently not recommended. Notably, a study has reported a low mortality rate of 1 in 10 HLH cases treated with tocilizumab (44). However, due to financial constraints, the patient declined both tocilizumab and immunoglobulin treatments. Ultimately, the patient succumbed to HLH within 16 days of diagnosis.

While previous reports have associated irAE-related HLH with pre-existing autoimmune disorders, including ankylosing spondylitis, psoriasis, and Sjögren’s syndrome (44–47), this represents the first instance of irAE-related HLH occurring in conjunction with SLE. The patient’s unsuccessful treatment outcome was attributed to the rapid progression of the disease, exacerbated by an excessive number of precursors, and further complicated by the patient’s non-compliance, including the refusal of timely evaluations and the use of a cytokine inhibitor proven effective against cytokine storms. In conclusion, the coexistence of MSI-H tumors and pre-existing SLE may exacerbate the prognose of irAE-related HLH, suggesting a potential interplay between these factors. Clinicians should maintain heightened vigilance regarding the potential emergence of HLH in patients with MSI-H cancers and SLE undergoing ICIs therapy. It is crucial to promptly initiate a combination of other pharmacological agents and steroid therapy upon the onset of HLH.

## Data Availability

The original contributions presented in the study are included in the article/supplementary material. Further inquiries can be directed to the corresponding author.
